# A new method for community-based intelligent screening of early Alzheimer’s disease populations based on digital biomarkers of the writing process

**DOI:** 10.3389/fncom.2025.1564932

**Published:** 2025-06-04

**Authors:** Shuwu Li, Kai Li, Jiakang Liu, Shouqiang Huang, Chen Wang, Yuting Tu, Bo Wang, Pengpeng Zhang, Yuntian Luo, Yanli Zhang, Tong Chen

**Affiliations:** ^1^School of Medical Technology and Information Engineering, Zhejiang Chinese Medical University, Hangzhou, China; ^2^School of Information Engineering, Hangzhou Medical College, Hangzhou, China; ^3^Zhejiang Engineering Research Center for Brain Cognition and Brain Diseases Digital Medical Instruments, Hangzhou Medical College, Hangzhou, China; ^4^Department of Neurology, Second Medical Center of Chinese PLA General Hospital, Beijing, China

**Keywords:** mild cognitive impairment, Alzheimer’s disease, digital biomarkers, early warning, writing

## Abstract

**Background:**

In response to the shortcomings of the current Alzheimer’s disease (AD) early populations assessment, which is based on neuropsychological scales with high subjectivity, low accuracy of repeated measurements, tedious process and dependence on physicians, it was found that digital biomarkers based on the writing process can effectively characterize the cognitive deficits of patients with mild cognitive impairment (MCI) due to AD.

**Methods:**

This study designed a digital writing assessment paradigm, extracted dynamic handwriting and image data during the paradigm assessment process, and analyzed digital biomarkers of the writing process to assess subjects’ cognitive functions. A total of 72 subjects, including 34 health controls (HC) and 38 MCI due to AD, were enrolled in this study.

**Results:**

Their combined screening efficacy of digital biomarkers based on the MCI writing process due to AD populations having an area under curve (AUC) of 0.918, and a confidence interval (CI) of 0.854–0.982, was higher than the Montreal Cognitive Assessment Scale (AUC = 0.859, CI = 0.772–0.947) and the Mini-mental State Examination Scale (AUC = 0.783, CI = 0.678–0.888).

**Conclusion:**

Therefore, digital biomarkers based on the writing process can characterize and quantify the cognitive function of MCI due to AD populations at a fine-grained level, which is expected to be a new method for intelligent screening and early warning of early AD populations in a community-based physician-free setting.

## 1 Introduction

Alzheimer’s disease (AD) is a progressive neurodegenerative disorder of the central nervous system, primarily characterized by a decline in cognitive function (Knopman et al., 2021). Currently, the course of AD is viewed as a continuum (Jack et al., 2018), with mild cognitive impairment (MCI) being an early stage of AD. MCI is mainly characterized by a mild decline in cognitive function and a high risk of progression to AD (McGrattan et al., 2022). It is estimated that there are currently about 44 million AD patients worldwide (Jia et al., 2020), and the probability of MCI converting to AD dementia within 3 years is as high as 55% (Okello et al., 2009). AD has an insidious onset and poor therapeutic effect after developing into the middle and late stages, which brings a heavy burden to the patient’s family and social economy (Chen et al., 2024). Therefore, focusing on the critical window for early detection and warning of AD, namely the MCI stage, is of great significance for the early prevention and treatment of AD.

Currently, there are two main modalities for clinically detecting MCI. One is amyloid β-protein (Aβ) pathological testing, including Aβ positron emission tomography (Aβ-PET) and cerebrospinal fluid testing (Leuzy et al., 2020; Burnham et al., 2019). However, current public awareness of MCI is low, and high-cost, invasive diagnostic methods are significantly reducing the rate of early diagnosis, which is not conducive to large-scale screening efforts. The other method is neuropsychological testing, such as the Mini-Mental State Examination (MMSE) and the Montréal Cognitive Assessment (MoCA) and other sets of assessment scales. However, neuropsychological tests are time-consuming, require a well-coordinated subject and an experienced clinician, and have highly subjective scores that are less accurate when repeated over a short period of time, and are easily influenced by the patient’s years of education (Yu et al., 2012). These factors greatly hinder the prevention and treatment process of AD. In particular, it is difficult to achieve objective and effective early warning of AD in places such as Asia and Africa, where populations are concentrated and medical care is unevenly distributed. Therefore, there is an urgent need to find new rapid and intelligent screening and early warning methods suitable for large-scale initial screening of MCI in community settings without physicians.

A large number of previous studies have assessed cognitive functioning in early AD populations, but there is a lack of time-series-based ratings of cognitive tasks to quantify cognitive functioning at a dynamic fine-grained level over the entire course of time. Numerous studies have shown that in the early stages of AD, patients exhibit equally significant deficits in several cognitive dimensions, such as executive functioning and speed of information processing (Hayden et al., 2012; Kim et al., 2021; Wu et al., 2023; Haworth et al., 2016). Executive functioning refers to the mental process by which an individual exerts conscious control over thoughts and behaviors, and involves a variety of higher-level cognitive abilities, such as inhibitory control, working memory, and cognitive flexibility (Diamond, 2013). Impairment of executive functioning in MCI due to AD is mainly manifested by decreased cognitive flexibility, impaired response inhibition, and decreased decision-making ability (Guarino et al., 2018). Information processing speed refers to the speed at which an individual goes from receiving information to making a response or decision, and this indicator plays an important role in cognitive functioning, learning ability, and daily life. The slower information processing speed in MCI due to AD is mainly manifested in brain sluggishness and delayed responses, requiring more time for the brain to process information (Haworth et al., 2016). Therefore, based on the above cognitive impairment characteristics, precise quantitative assessment of executive function and information processing speed in patients with MCI due to AD can provide insights for early screening and warning.

It has been found that physiological and behavioral “digital biomarkers” captured by digital devices can make up for the shortcomings of traditional diagnostic methods, which are dependent on clinicians, subjective, and difficult to quantify digitally, because of their objective, quantifiable, and fine-grained portrayal of complex processes (Lio et al., 2019). Currently, digital biomarkers have received more and more attention from scholars in the field of early screening and early warning of AD (Bera et al., 2019; Lio et al., 2019). In recent years, digital biomarkers based on writing characteristics have shown good potential for early screening for AD. Writing activity, as a complex executive process, requires the joint participation of cognitive, kinesthetic, and perceptual-motor components (Tseng and Cermak, 1993). Characteristics of writing behavior during a writing task not only effectively reflect the participant’s executive function, but also adequately reflect multiple domains of cognitive function, such as higher-order neurocognitive planning and speed of information processing (Piers et al., 2017). Jacek Kawa extracted digital biomarkers based on short and long text data to screen and identify MCI (Kawa et al., 2017). Nan-Ying Yu extracted digital handwriting biomarkers based on computerized handwriting to further identify MCI (Yu and Chang, 2019). Kai Li extracted digital biomarkers of fingertip-interactional writing to analyze the impairment of spatial executive process in people with MCI due to AD, achieving area under curve (AUC) of 0.88 (Li et al., 2023). Thus, digital biomarkers based on the assessment paradigm are expected to be an effective way to screen and warn people in the early stages of AD.

To summarize, we hypothesized that digital biomarkers of the writing process can be used to characterize cognitive deficits in people with MCI due to AD at a fine-grained level. To test the above hypothesis, we developed a digital writing assessment paradigm to extract cognitive deficits in executive function and information processing speed characteristic of MCI due to AD, and provide new ideas for intelligent and rapid screening and early warning of early AD patients in a community-based physician-free setting.

## 2 Materials and methods

### 2.1 Human-computer interaction paradigm design and experimental procedures

We hypothesized that digital biomarkers of the writing process hold promise for fine-grained portrayal of cognitive deficits in MCI due to AD populations. A digital writing evaluation paradigm was designed using projected mutual capacitive tactile feedback technology.

#### 2.1.1 Experimental paradigm design hardware and software foundations

**Figure 1:**
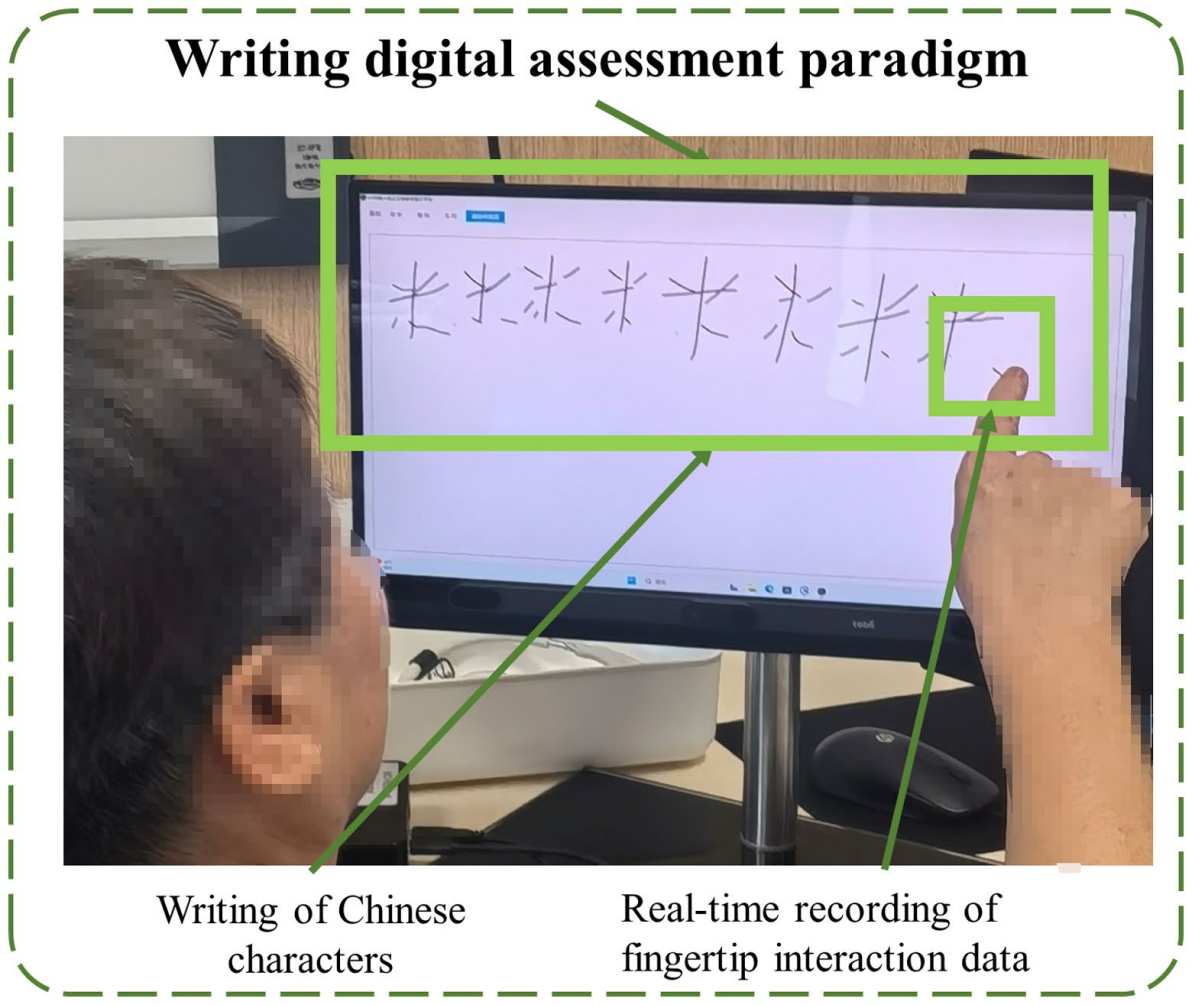
The schematic diagram of the paradigm.

The hardware requirements for this experiment include an Intel computer (NUC11PAHi5), and a 3,840*2,160 pixel touchable monitor (length, width and height: 392*250*10 mm, 17.3 in.). The software system involved in this experiment is based on the pre-existing human-computer interaction system (Tao et al., 2023). The writing digital assessment paradigm is built using HTML5 Canvas, which in turn meets the software requirements for the experiment. A schematic diagram of the paradigm is shown in Figure 1. This experiment collected behavioral data from participants (specifically, the trajectory of writing the Chinese character “米”) and did not gather any personally identifiable information, such as facial data. All behavioral data were uniformly uploaded to our local database, where they were stored under anonymised identifiers. A dedicated database engineer managed and maintained the data to minimize the risk of breaches.

#### 2.1.2 Experimental paradigm design and principle interpretation

There is a close relationship between writing and executive functions and speed of information processing. Information processing speed is the foundation of writing fluency, and executive functions ensure the organization and accuracy of writing through planning and regulation, and both work together to influence writing efficiency and writing results. Chinese characters for “米” as a handwriting assessment task, it is mainly based on its unique advantages in the Chinese writing system: (1) Operational simplicity (few strokes and clear structure), (2) Age-friendliness (high frequency character, reducing cognitive load), and (3) Cultural universality (highly related to daily life in terms of semantics, with low dependence on years of education). These characteristics make it an ideal tool for cognitive screening in the Chinese population (Li et al., 2023). The specific design of the writing digital assessment paradigm in this study was as follows: subjects are required to write 10 Chinese characters for “米” on the screen through fingertip interaction, and the time limit of the paradigm is 3 min.

#### 2.1.3 Definition and quantitative analysis of digital biomarkers in the writing process

Based on the above raw data and pre-existing algorithms (Li et al., 2023), we extracted digital biomarkers of the writing process via python 3.10.0. In order to portray the cognitive functions of the subjects at a fine-grained level, we categorized the digital biomarkers into information processing speed digital biomarkers and executive function digital biomarkers.

The information processing speed digital biomarkers (*IPSDB*) measured subjects’ planning and thinking before execution through fingertip interaction, including total task time (*IPSDB*_1_), task process time (*IPSDB*_2_), writing time (*IPSDB*_3_), total pause time (*IPSDB*_4_), initial pause time (*IPSDB*_5_), total process pause time (*IPSDB*_6_), maximum process pause time (*IPSDB*_7_), average process pause time (*IPSDB*_8_), variability in process pause time (*IPSDB*_9_), and the number of pauses (*IPSDB*_10_). The total task time was the time interval from when subjects entered the paradigm to when they finished writing the 10 Chinese characters for “米,” i.e., the total time taken to complete the paradigm. The task time is the time interval from when the subjects started writing to when they finished writing the 10 Chinese characters for “米.” The writing time was the time the subject’s finger wrote on the screen during the paradigm evaluation. Total pause time was the time when the subject’s finger did not touch the screen during the paradigm assessment, which was used to characterize the subject’s thinking throughout the writing process. The initial pause time was the time between the subject’s entry into the paradigm and the first start of writing, and it was used to characterize the subject’s starting thinking time. Total process pause time, maximum process pause time, average process pause time, variability in process pause time, and the number of pauses were used to characterize the subject’s process-planning thinking after the subject started writing. Research indicates that patients with early-stage Alzheimer’s disease (AD) may experience prefrontal metabolic decline, which affects their ability to set goals, sequence tasks, and self-monitor during writing, leading to increased writing task duration (Schroeter et al., 2012; Jagust, 2018). Neuroimaging studies have identified cortical thinning and amyloid deposition in the prefrontal cortex of AD patients, which may impair motor coordination and semantic processing required for writing (Liu et al., 2022). Additionally, the dorsolateral prefrontal cortex (DLPFC), a subregion of the prefrontal cortex located in the lateral frontal lobe, plays a central role in executive functions such as working memory, decision-making, and attentional control (Jung et al., 2022; Hagler and Sereno, 2006; Lüthi et al., 2014). In this study, abnormalities in working memory (e.g., Chinese character writing) and spatial planning (e.g., character structure) were found to indicate an early risk of Alzheimer’s disease.

**Figure 2:**
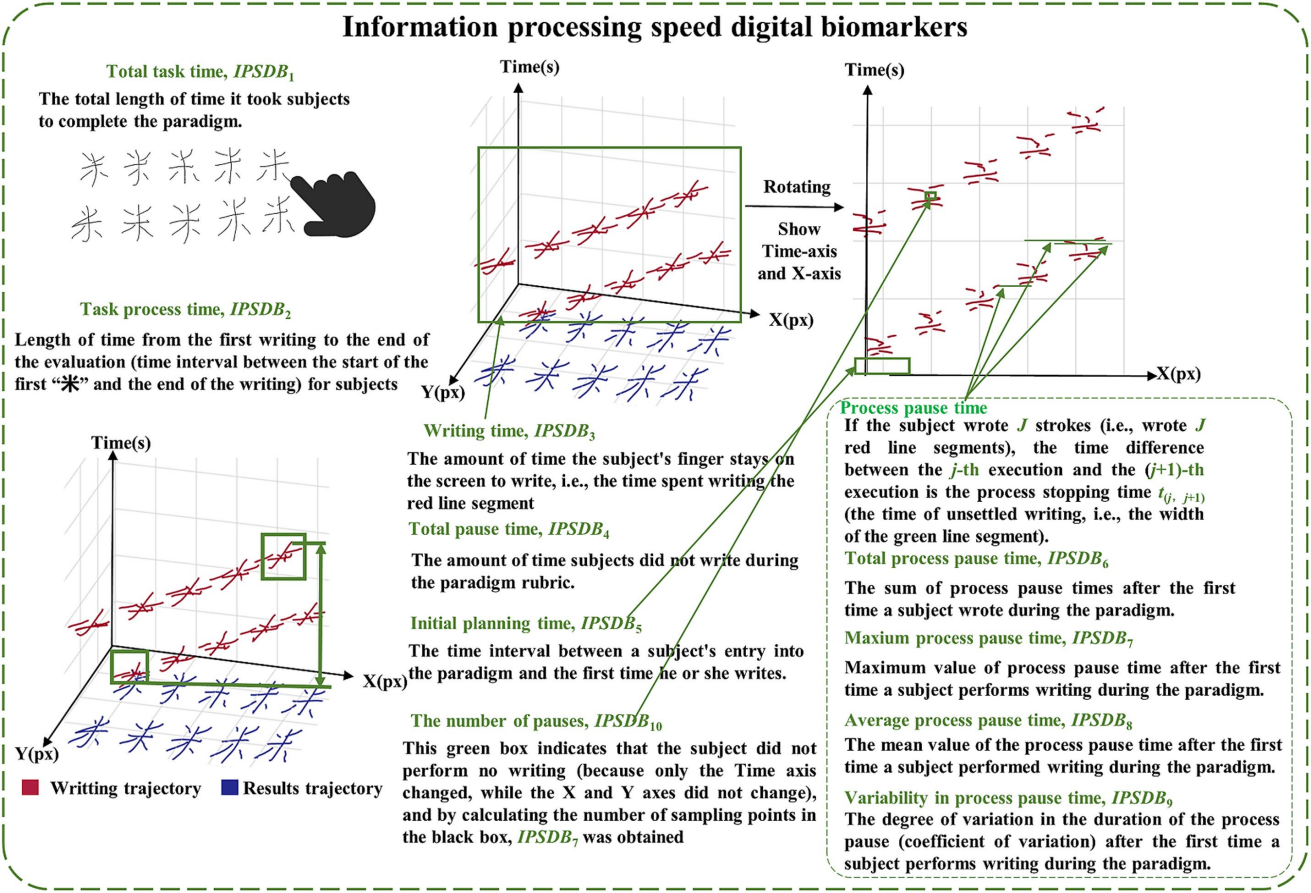
Illustration of information processing speed digital biomarkers.

**Figure 3:**
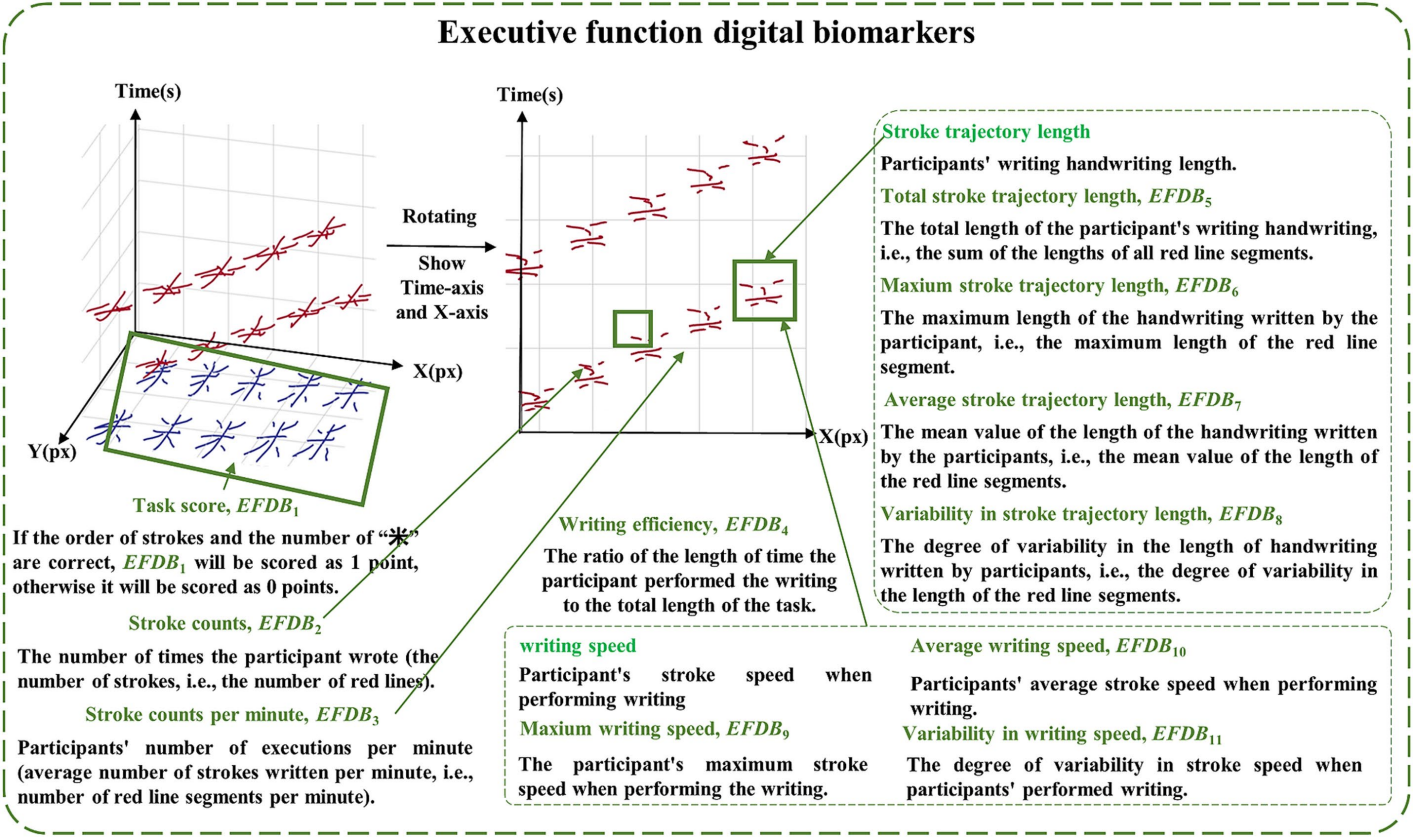
Illustration of executive function digital biomarkers.

The executive function digital biomarkers (*EFDB*) was a fingertip interactive technology that measures the subject’s performance after “thinking,” including task score (*EFDB*_1_), stroke counts (*EFDB*_2_), stroke counts per minute (*EFDB*_3_), writing efficiency (*EFDB*_4_), total stroke trajectory length (*EFDB*_5_), maximum stroke trajectory length (*EFDB*_6_), average stroke trajectory length (*EFDB*_7_), variability in stroke trajectory length (*EFDB*_8_), maximum writing speed (*EFDB*_9_), average writing speed (*EFDB*_10_) and variability in writing speed (*EFDB*_11_). Among them, the task score was a rating of the subjects’ writing process and the writing result, which was used to judge whether the order of the strokes in writing the Chinese characters for “米” was correct or not, and whether the number of Chinese characters for “米” was correct or not after the completion of the writing. The stroke count was the number of times a subject’s finger paused while writing on the screen during the paradigm assessment. The stroke count per minute was the number of strokes written per minute during the paradigm assessment. Research indicates that the lateral prefrontal cortex is responsible for representing and selecting task rules, which may affect the planning of stroke order and structure. For example, patients with frontal damage may exhibit executive dysfunction, making it difficult to effectively control stroke numbers (Shimamura, 2000). Moreover, the prefrontal cortex maintains writing rules (e.g., stroke order in Chinese characters) through working memory, thereby reducing redundant strokes (Han et al., 2009; Collette et al., 2005). Writing efficiency was the cumulative length of time the subject’s finger spent writing on the screen during the paradigm evaluation process, and then analyzing that length as a percentage of task length. Research shows that the prefrontal cortex (especially the right prefrontal cortex) exhibits increased activity during predictive tasks and is linked to executive planning abilities. This function has a direct impact on writing fluency, which in turn affects writing efficiency (Newman et al., 2003). Total stroke trajectory length, maximum stroke trajectory length, average stroke trajectory length, and variability in stroke trajectory length were used to characterize the stroke trajectories of the finger writing on the screen during the paradigm evaluation process of the subjects. Maximum writing speed, average writing speed, and variability in writing speed were used to characterize the stroke speed of finger writing on screen during the paradigm assessment. Research shows that individuals with mild cognitive impairment (MCI) and Alzheimer’s disease (AD) exhibit significantly slower writing speeds during dictation tasks compared to healthy controls (HC). This may be related to cognitive deficits caused by damage to the temporal cortex (An et al., 2023). To facilitate future analysis of digital biomarkers mining, we provided detailed conceptual definitions of various digital biomarkers in the paradigm, and digital biomarker illustrations are shown in Figures 2, 3. Digital information processing speed biomarkers is shown in Table 1, and digital executive function biomarkers is shown in Table 2.

**Table 1:** Information processing speed digital biomarkers.

Cognitive functioning deficit	Digital biomarkers	Abbreviation	Interpretation and units
Decreased speed of information processing may be present in patients with early stages of AD (Shi et al., 2020)	Information processing speed digital biomarkers _1_: total task time	*IPSDB*_1_	It was used to calculate the length of the task from the time the subjects enters the paradigm to the time of subjects finishes writing 10 Chinese characters for “米,” i.e., the total time taken to complete the paradigm. (Seconds, s)
	Information processing speed digital biomarkers_2_: task process time	*IPSDB*_2_	It was used to calculate the length of time it takes for a subject to finish writing 10 Chinese characters for “米” after the first writing session during the paradigm assessment. (Seconds, s)
	Information processing speed digital biomarkers_3_: writing time	*IPSDB*_3_	It was used to calculate the length of time the subject’s finger was writing on the screen during the paradigm assessment. (Seconds, s)
	Information processing speed digital biomarkers_4_: total pause time	*IPSDB*_4_	It was used to calculate the amount of time the subject’s finger was not in contact with the screen during the paradigm rubric. (Seconds, s)
	Information processing speed digital biomarkers_5_: initial pause time	*IPSDB*_5_	It was used to calculate the time between when a subject enters the paradigm and when they first start writing. (Seconds, s)
	Information processing speed digital biomarkers_6_: total process pause time	*IPSDB*_6_	It was used to calculate the time between when a subject enters the paradigm and when they first start writing. (Seconds, s) It was used to calculate the total time period during which the subject’s finger did not touch the screen after first starting to write. (Seconds, s)
	Information processing speed digital biomarkers_7_: maximum process pause time	*IPSDB*_7_	It was used to calculate the maximum time a subject’s finger was not in contact with the screen after the first writing session during the paradigm assessment. (Seconds, s)
	Information processing speed digital biomarkers_8_: average process pause time	*IPSDB*_8_	It was used to calculate the average time that a subject’s finger did not touch the screen after the first writing session during the paradigm evaluation. (Seconds, s)
	Information processing speed digital biomarkers_9_: variability in process pause time	*IPSDB*_9_	It was used to calculate the degree of change (coefficient of variation) in the length of time a subject’s finger was not in contact with the screen after the first writing session during the paradigm rubric.
	Information processing speed digital biomarkers_10_: the number of pauses	*IPSDB*_10_	This indicator is used to count the number of finger pauses when the subject’s finger is written on the screen during the paradigm assessment. (Times)

**Table 2:** Executive function digital biomarkers.

Cognitive functioning deficit	Digital biomarkers	Abbreviation	Interpretation and units
Possible decline in executive function in patients with early AD (Wu et al., 2023).	Executive function digital biomarkers_1_: task score	*EFDB*_1_	It was used for the writing process and the writing result of the subjects to determine whether the order of the strokes in writing the Chinese characters for “米” was correct or not, and whether the Chinese characters for “米” Chinese characters was correct or not after the completion of the writing, and to calculate the decision-making performance of the subjects. (Score)
	Executive function digital biomarkers_2_: stroke counts	*EFDB*_2_	It was used to count the number of finger pauses when the subject used their finger to write on the screen during the paradigm assessment. (Times)
	Executive function digital biomarkers_3_: stroke counts per minute	*EFDB*_3_	It was used to calculate the number of strokes written by the subject during the paradigm assessment per minute for calculation. (Times)
	Executive function digital biomarkers_4_: writing efficiency	*EFDB*_4_	It was used to calculate the cumulative length of time the subject’s finger was writing on the screen during the paradigm assessment, and then analyze that length as a percentage of task length, i.e., execution efficiency.
	Executive function digital biomarkers_5_: total stroke trajectory length	*EFDB*_5_	It was used to calculate the maximum value of the stroke trajectory length of a subject’s finger writing on the screen during paradigm evaluation. (Pixels, px)
	Executive function digital biomarkers_6_: maximum stroke trajectory length	*EFDB*_6_	It was used to calculate the maximum value the of the stroke trajectory length of a subject’s finger writing on the screen during paradigm evaluation. (Pixels, px)
	Executive function digital biomarkers_7_: average stroke trajectory length	*EFDB*_7_	It was used to calculate the average length of the stroke trajectory of a subject’s finger writing on the screen during the paradigm assessment. (Pixels, px)
	Executive function digital biomarkers_8_: variability in stroke trajectory length	*EFDB*_8_	It was used to calculate the degree of variability in the stroke trajectory length of a subject’s finger writing on the screen during paradigm assessment, i.e., the variability in stroke trajectory length.
	Executive function digital biomarkers_9_: maximum writing speed	*EFDB*_9_	It was used to calculate the maximum stroke speed when the subject’s finger was written on the screen during the paradigm evaluation. (Pixels/Seconds, px/s)
	Executive function digital biomarkers_10_: average writing speed	*EFDB*_10_	It was used to calculate the average stroke speed of the subject’s finger while writing on the screen during the paradigm evaluation. (Pixels/Seconds, px/s)
	Executive function digital biomarkers_11_: variability in writing speed	*EFDB*_11_	It was used to calculate the degree of variability in stroke speed when the subject used their finger to write on the screen during the paradigm assessment.

We know the total task time (*IPSDB*_1_), the initial pause time (*IPSDB*_5_) and the stroke counts (*EFDB*_2_) of a certain subject, who has written a total of *J* strokes (*EFDB*_2_ = *J*), the time interval between the *j*-th stroke and the (*j* + 1)-th stroke is *t*_(*j*, *j* + 1)_, and the time of the *j*-th stroke writing was *t_j_*, and there are a total of *K* stroke coordinate points in the process, the *k*-th stroke coordinate point is (*X_k_*, *Y_k_*). The task process time (*IPSDB*_2_) is calculated by Equation 1, the total pause time (*IPSDB*_4_) is calculated by Equation 2, the total process pause time (*IPSDB*_6_) is calculated by Equation 3, the maximum process pause time (*IPSDB*_7_) is calculated by Equation 5, the average process pause time (*IPSDB*_8_) is calculated by Equation 4 and the variability in process pause time (*IPSDB*_9_) is calculated by Equation 7, the specific details are as follows:


(1) $$\text{IPSD}B_{2}=\text{IPSD}B_{1}-\text{IPSD}B_{5}$$

(2) $$\text{IPSD}B_{4}=\text{IPSD}B_{5}+\text{IPSD}B_{6}$$

(3) $$\text{IPSD}B_{6}=\sum_{j=1}^{J-1}t_{\left(j,j+1\right)}(1\leq j\leq J,j\in N)$$

(4) $$\text{IPSD}B_{8}=\frac{\text{IPSD}B_{6}}{J-1}$$

(5) $$\text{IPSD}B_{7}=\max_{1\leq j\leq J}t_{\left(j,j+1\right)}$$

(6) $$\sigma_{t}=\sqrt{\frac{\sum_{j=1}^{J-1}{\left(t_{\left(j,j+1\right)}-\text{IPSD}B_{8}\right)}^{2}}{J-1}}$$

(7) $$\text{IPSD}B_{9}=\frac{\sigma_{t}}{\text{IPSD}B_{8}}$$

In Equation 6, $\sigma_{t}$ was the standard deviation of the process pause length.

The writing time (*IPSDB*_3_) is calculated by Equation 8, the number of pauses (*IPSDB*_10_) is calculated by Equation 10, the stroke counts per minute (*EFDB*_3_) is calculated by Equation 11, the writing efficiency (*EFDB*_4_) is calculated by Equation 12, the total stroke trajectory length (*EFDB*_5_) is calculated by Equation 14, the maximum stroke trajectory length (*EFDB*_6_) is calculated by Equation 15, and the average stroke trajectory length (*EFDB*_7_) is calculated by Equation 16, the specific details are as follows:


(8) $$\text{IPSD}B_{3}=\sum_{j=1}^{J}t_{j}(1\leq j\leq J,j\in N)$$

(9) $$\text{pause}(j,k)=\{\begin{array}{c} 1\;(X_{k+1}=X_{k}\;\text{and}\;Y_{k+1}=Y_{k})\\ 0\;\;(X_{k+1}\neq X_{k}\;\text{or}\;Y_{k+1}\neq Y_{k}) \end{array}(1\leq k\leq K-1,k\in N)$$

(10) $$\text{IPSD}B_{10}=\sum_{j=1}^{J-1}\sum_{k=1}^{K-1}\text{pause}(j,k)$$

(11) $$\mathrm{EFD}B_{3}=\frac{\mathrm{EFD}B_{2}}{\text{IPSD}B_{1}}*60(\mathrm{EFD}B_{2}=J)$$

(12) $$\mathrm{EFD}B_{4}=\frac{\text{IPSD}B_{3}}{\text{IPSD}B_{1}}$$

(13) $$D_{j}=\sum_{k=1}^{K-1}\sqrt{{\left(X_{k+1}-X_{k}\right)}^{2}+{\left(Y_{k+1}-Y_{k}\right)}^{2}}$$

(14) $$\mathrm{EFD}B_{5}=\sum_{j=1}^{J-1}D_{j}$$

(15) $$\mathrm{EFD}B_{6}=\max_{1\leq j\leq J}t_{j}$$

(16) $$\mathrm{EFD}B_{7}=\frac{\mathrm{EFD}B_{5}}{J}$$

In Equation 9, *pause*(*j*,*k*) was used to determine whether the *k*-th stroke coordinate point and the (*k* + 1)-th stroke coordinate point are the same when the subject draws the *j*-th stroke. In Equation 13, *D_j_* was the trajectory length of the *j*-th stroke. The variability in stroke trajectory length (*EFDB*_8_) is the same as the variability in process pause time (*IPSDB*_9_). The maximum writing speed (*EFDB*_9_) is calculated by Equation 17, the average writing speed (*EFDB*_10_) is calculated by Equation 18, and the variability in writing speed (*EFDB*_11_) is calculated by Equation 20, the specific details are as follows:


(17) $$\mathrm{EFD}B_{9}=\max_{1\leq j\leq J}\sum_{j=1}^{J-1}\frac{D_{j}}{t_{j}}$$

(18) $$\mathrm{EFD}B_{10}=\frac{\sum_{j=1}^{J-1}\frac{D_{j}}{t_{j}}}{J}$$

(19) $$\sigma_{v}=\sqrt{\frac{\sum_{j=1}^{J-1}{\left(t_{\left(j,j+1\right)}-\mathrm{EFD}B_{10}\right)}^{2}}{J-1}}$$

(20) $$\mathrm{EFD}B_{11}=\frac{\sigma_{v}}{\mathrm{EFD}B_{10}}$$

In Equation 19, $\sigma_{v}$ was standard deviation of writing speed.

#### 2.1.4 Design of experimental rules

The experiment was conducted in a quiet room to avoid interference with the results from ambient noise. We placed a comfortable and stable chair in front of the touch screen display. Staff assisted participants in adjusting their posture and position to maintain a distance of approximately 30 centimeters from the touch screen display. This design ensured that the subjects could clearly observe the content of the screen and easily manipulate the screen with their fingers, thus avoiding any adverse effects on the experiment due to visual impairment or manipulation difficulties.

### 2.2 Experimental setup

#### 2.2.1 Estimated sample size

In this study, the sample size for final inclusion was roughly estimated by the G*Power tool, with the relevant parameters as ‘Test family’ choosing ‘t tests’, for ‘Statistical test’ select ‘Means: Difference between two independent means (two groups)’, for ‘Tail(s)’ select ‘Two’, for ‘Type of power analysis’, select ‘*A priori*: Compute required sample size - given *α*, power, and effect size’, “Effect size d” is “0.7,” ‘α err prob’ fill in “0.05,” “Power(1 - β err prob)” fill in “0.8,” “Allocation ratio N2/N1” fill in “1,” and the total sample size was calculated to be 68, 34 persons for each of the healthy control (HC) population, and 34 persons for persons with MCI due to AD.

#### 2.2.2 Subjects

A total of 75 subjects were recruited for this study at the First Medical Centre of the General Hospital of the Chinese People’s Liberation Army. Diagnostic criteria for MCI due to AD origin were consistent with the 2011 National Institute on Aging and Alzheimer’s Disease Association (NIA-AA) criteria (Albert et al., 2011). The healthy control (HC) populations had no subjective complaints or objective evidence of neurological disorders, and all diagnoses were made by two experienced neurologists. Specific criteria for this study were as follows:

Inclusion criteria for MCI due to AD: (1) NIA-AA criteria were met and subjective memory loss was present; (2) Minimum Mental State Examination (MMSE) total score >24; (3) no significant deficits in ability to perform activities of daily living and Clinical Dementia Rating (CDR) score of 0.5; (4) Ability to cooperate in doing the examination as well as the paradigm tasks, right-handedness; (5) Participants were required to have a primary school or higher education level.

Exclusion criteria for MCI due to AD: (1) Atypical forms of AD, such as the posterior cortical variant, progressive aphasia variant, frontal variant, and Down’s syndrome; (2) Severe psychiatric conditions like major depression, cerebrovascular disease, metabolic disorders, central nervous system infections, intracranial tumors, or severe cardiac, hepatic, pulmonary, or renal diseases; (3) Conditions affecting clinical assessment, such as motor impairments from Parkinson’s disease or arthritis, aphasia, severe auditory or visual impairments, or severe dysarthria.

Inclusion criteria for HC: HC were volunteers or healthy spouses of patients who came to the hospital for health checkups during the same period, (1) Without complaints of memory loss or other cognitive decline, and no history of cognitive impairment; (2) MMSE scores above the cut-off score >17 points for those with elementary school education and below, and >24 points for those with junior high school education and above); daily life function scale (activity of daily life, ADL) ≤ 20 points; (3) CDR-global = 0 points; (4) Able to cooperate in doing the examination as well as the paradigm tasks, right-handedness. Daily life, ADL) ≤ 20 points; (5) Participants were required to have an educational level of primary school or higher.

All subjects were native Chinese speakers and long-term residents of China. They provided informed consent before considering inclusion. During the conduct of the formal trial, two people in the MCI group refused to participate due to flu and one person in the HC group withdrew from the trial for other reasons. A total of 38 cases of MCI of AD origin and 34 cases of HC were finally included in this study. The study followed the basic principles of the Helsinki Declaration, an international code of medical ethics, and was approved by the Medical Ethics Committee of the General Hospital of the Chinese People’s Liberation Army (Ethics No. S2022-770-02). Clinical information about each patient was collected, including demographic data, general clinical information, and neuropsychological assessments, including MMSE and MoCA, were performed on all subjects. Standardized Chinese versions of the MMSE and MoCA were used (Jia et al., 2021).

### 2.3 Paradigm experimental process

**Figure 4:**
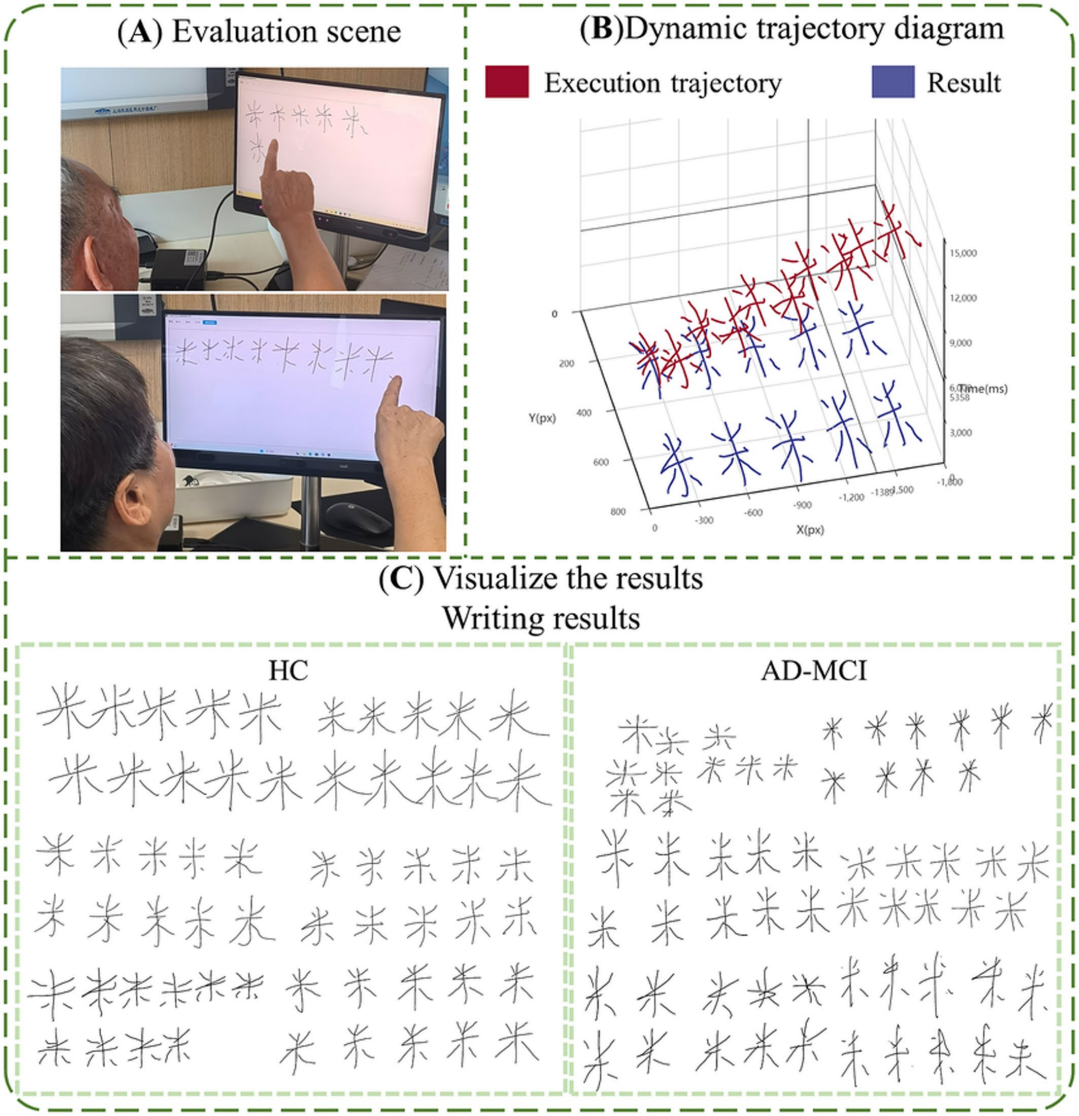
The information of paradigm. **(A)** Evaluation scene; **(B)** visualize the results, dynamic trajectory diagram; **(C)** visualize the results, writing results.

Based on the research hypothesis that digital biomarkers through the writing process are expected to portray cognitive deficits at a fine-grained level in MCI due to AD populations, we designed a writing digital assessment paradigm for assessing cognitive function. Before the paradigm began, we trained staff in advance, informing them about the testing process and the operation of the human-computer interaction system. Subsequently, the staff would inform the participants about the standard procedure and handling methods. All subjects were in a quiet room for the assessment. A comfortable and stable chair was placed in front of the touch screen display. Staff helped participants adjust their posture and position to about 30 cm from the display, ensuring easy access to the screen. Subjects were asked to write continuously on the touch screen with the tip of their right index finger. Subjects had only one chance to perform the writing-number evaluation paradigm. If a subject’s task duration exceeded 3 min, they were considered unable to complete the paradigm and were excluded from subsequent analyses. We used an artificial intelligence algorithm to extract digital biomarkers of the writing process, and then assessed the subject’s cognitive deficits, and professional clinicians will give patients corresponding suggestions based on clinical evaluation results and paradigm evaluation results to reduce the potential impact of paradigm evaluation results on patients’ health, and the paradigm information is shown in Figure 4.

### 2.4 Statistical analysis

All statistical analyses were performed using the generalized data analysis software package SPSS 26.0. The count data were compared with the chi-square test to compare the differences between groups, and the measurement data conforming to the normal distribution were expressed as x ± s, and the t-test independent samples were used to compare the differences between groups; the measurement data conforming to the skewed distribution were expressed as M(IQR), and the Mann–Whitney U test was used to compare the differences between groups. Second, we used binary logistic stepwise regression to screen digital biomarkers of the writing process. Finally, we plotted the subjects’ work characteristic curve (ROC) and determined the single digital writing process biomarker and multiple digital writing process biomarkers combined to alert the MCI group by comparing the area under the curve. *p* < 0.05 was considered statistically significant.

## 3 Results

### 3.1 Demographic and clinical characteristics

**Figure 5:**
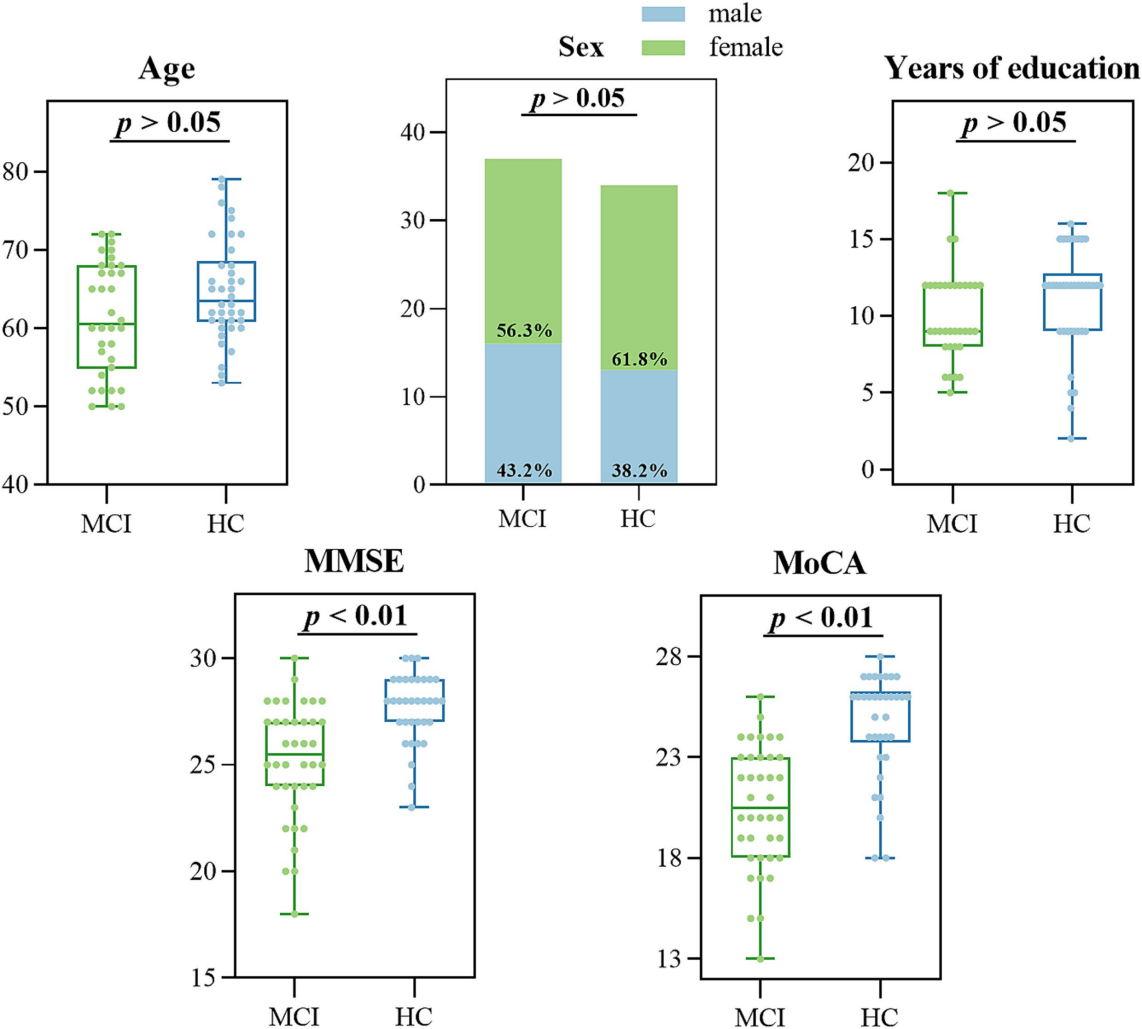
Data distribution of demographic and clinical characteristics in the MCI and HC groups.

The effective sample size of this study was 72 participants, 38 MCI due to AD and 34 HC, who were included in the MCI and HC groups, respectively. We analyzed the baseline information of the two groups for differences, and there were no significant differences (*p* > 0.05) between the two groups of participants in age (*p* = 0.068), gender (*p* = 0.738), and years of education (*p* = 0.062), as shown in Figure 5 and Table 3. Participants’ cognitive functioning was assessed by clinicians at the time of enrolment, and we performed the MoCA, MMSE Neurological Psychological Scale, and an intergroup variability analysis, suggesting that both groups had significantly lower MMSE total scores (*p* < 0.001) and MoCA total scores (*p* < 0.001) in the MCI group than in the HC group. All *p*-values reported are two-tailed unless stated otherwise.

**Table 3:** Differential results of demographic and clinical characteristics in the MCI and HC groups.

	MCI *n* = 38	HC *n* = 34	*p* value
Age, years	63.50 (7.75)	60.50 (13.25)	0.068
Sex (female/male)	22/16	21/13	0.738
Years of education	12.00 (3.75)	9.00 (4.00)	0.062
MMSE score **	25.50 (3.00)	28.00 (2.00)	<0.001
MoCA score **	20.00 (5.00)	26.00 (2.50)	<0.001

** Indicates a significant difference between the two groups, at *p* < 0.01.

### 3.2 Analysis of digital biomarkers

**Figure 6:**
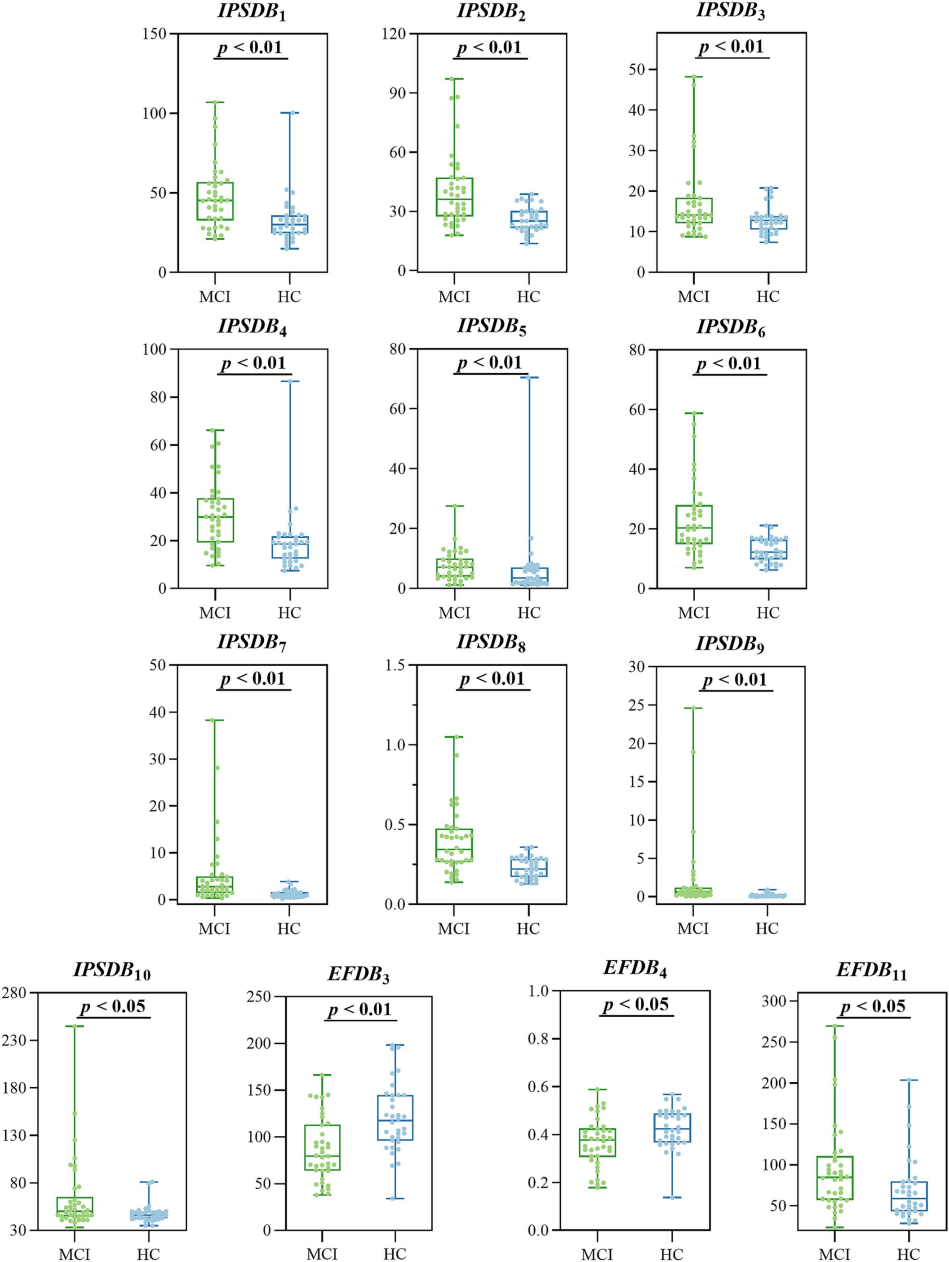
Digital biomarkers of the writing process with intergroup variability in the MCI and HC groups.

We analyzed the differences in digital biomarkers in the writing process between MCI and HC groups. The results showed that a total of 13 writing process digital biomarkers were significantly different between the HC and MCI groups (*p* < 0.05). In the MCI group, total task time (*IPSDB*_1_), task process time (*IPSDB*_2_), stroke counts per minute (*IPSDB*_3_), total pause time (*IPSDB*_4_), initial pause time (*IPSDB*_5_), total process pause time (*IPSDB*_6_), maximum process pause time (*IPSDB*_7_), average process pause time (*IPSDB*_8_), variability in process pause time (*IPSDB*_9_), the number of pauses (*IPSDB*_10_), and variability in writing speed (*EFDB*_11_) were significantly higher than that of the HC group, whereas the number of strokes counts per minute (*EFDB*_3_) and writing efficiency (*EFDB*_4_) were significantly lower than that of the HC group, and the results of the test of variability between the two groups are shown in Figure 6 and Tables 4, 5.

**Table 4:** Differential results of digital biomarkers of information processing speed in the MCI and HC groups.

	MCI *n* = 38	HC *n* = 34	*p* value
*IPSDB*_1_, s **	47.23 (27.91)	64.85 (41.70)	<0.001
*IPSDB*_2_, s **	36.17 (19.62)	25.09 (8.70)	<0.001
*IPSDB*_3_, s **	14.18 (6.32)	12.77 (3.28)	<0.001
*IPSDB*_4_, s **	29.94 (18.54)	18.57 (9.29)	<0.001
*IPSDB*_5_, s **	7.05 (6.11)	3.40 (5.23)	0.007
*IPSDB*_6_, s **	20.33 (13.20)	12.24 (6.67)	<0.001
*IPSDB*_7_, s **	2.73 (3.54)	1.00 (0.95)	<0.001
*IPSDB*_8_, s **	0.34 (0.21)	0.22 (0.11)	<0.001
*IPSDB*_9_, s **	0.61 (0.94)	0.11 (0.14)	<0.001
*IPSDB*_10_, time *	50.00 (20.5)	46.00 (7.25)	0.035

* Indicates a significant difference between the two groups, at *p* < 0.05; ** indicates a significant difference between the two groups, at *p* < 0.01.

**Table 5:** Differential results of digital biomarkers of executive function in MCI and HC groups.

	MCI *n* = 38	HC *n* = 34	*p* value
*EFDB*_1_, score	0.00 (1.00)	1.00 (1.00)	0.103
*EFDB*_2_, time	59.68 (6.26)	57.71 (3.71)	0.075
*EFDB*_3_, time **	79.65 ± 49.51	117.55 ± 48.87	<0.001
*EFDB*_4_*	0.38 (0.12)	0.42 (0.12)	0.011
*EFDB*_5_, px	9,051.29 ± 2,565.92	8,969.04 ± 2,186.12	0.146
*EFDB*_6_, px	417.12 (276.62)	388.04 (164.86)	0.752
*EFDB*_7_, px	154.49 ± 51.35	156.01 ± 38.44	0.889
*EFDB*_8_, px	57.48 (40.83)	48.90 (42.95)	0.122
*EFDB*_9_, px/s	1,068.36 (699.69)	1,131.05 (330.49)	0.423
*EFDB*_10_, px/s	594.18 ± 249.91	690.49 ± 160.21	0.059
*EFDB*_11_*	84.64 (53.68)	58.75 (36.45)	0.011

* Indicates a significant difference between the two groups, at *p* < 0.05; ** indicates a significant difference between the two groups, at *p* < 0.01.

### 3.3 ROC curves for identifying MCI due to AD patients from all participants

**Figure 7:**
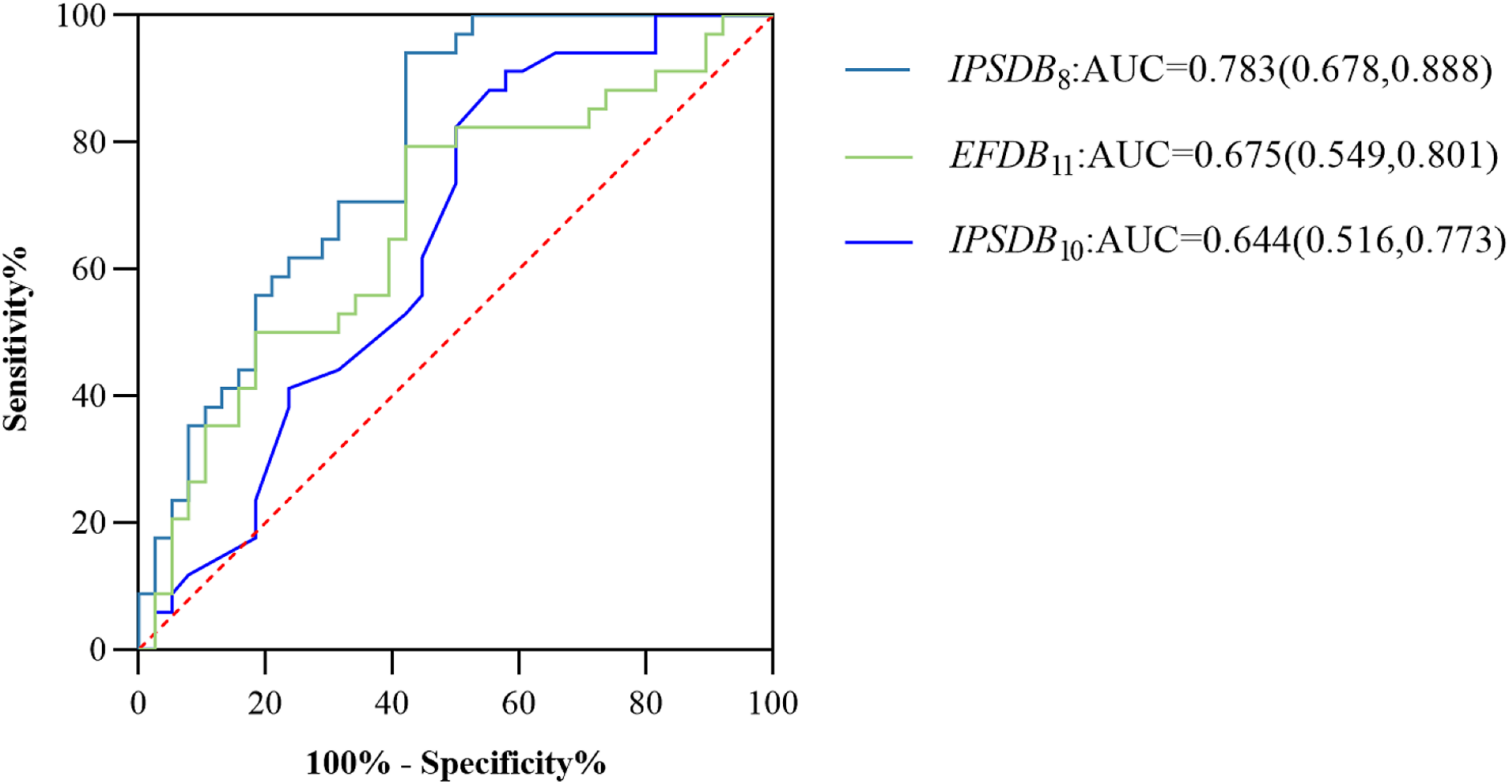
ROC curves, area under the curve, and 95% confidence intervals for the prediction of cognitive deficits for each of the 3 writing process digital biomarkers after stepwise regression for the MCI and HC groups of the populations.

**Figure 8:**
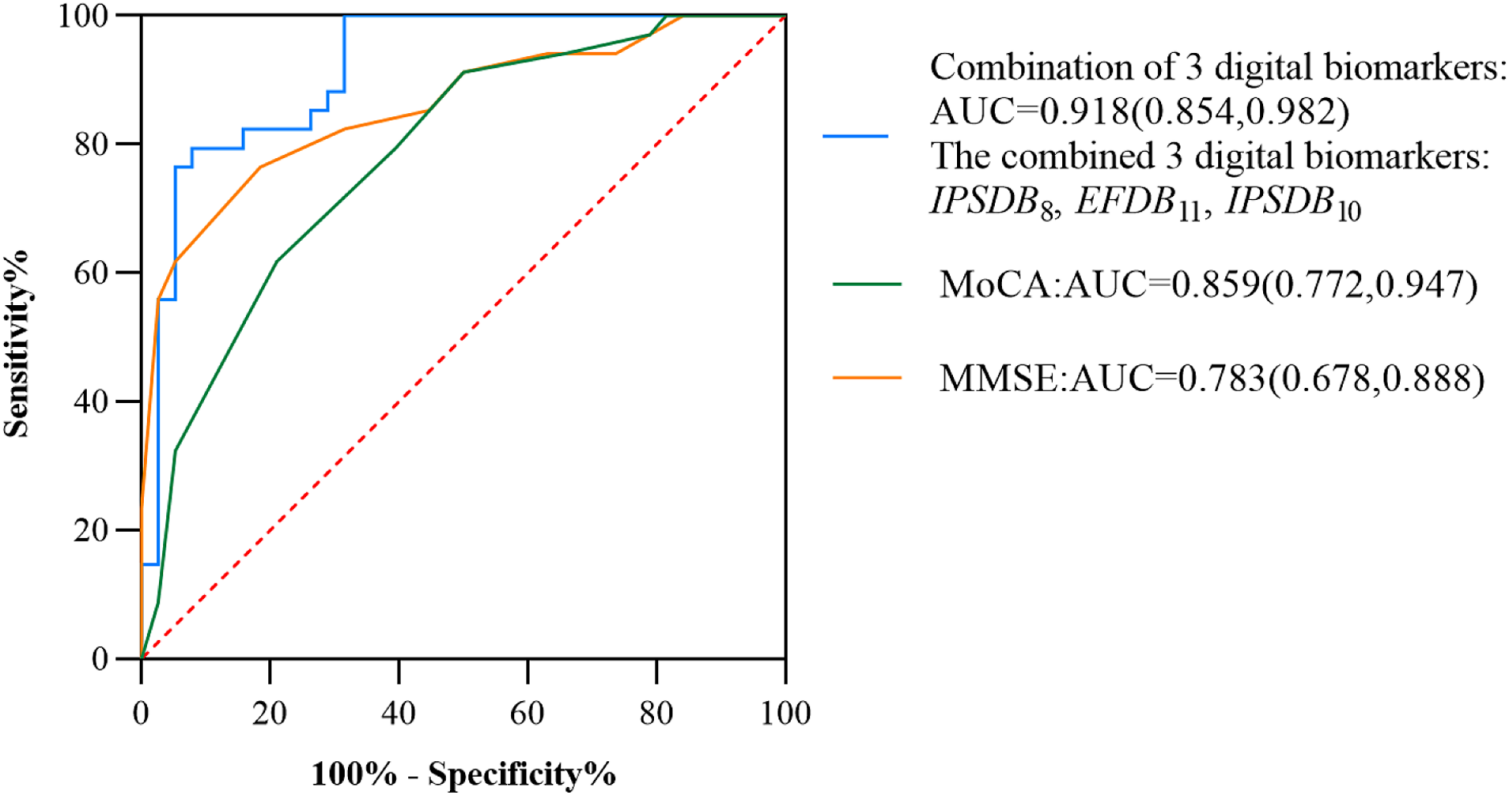
ROC curves, area under the curve, and 95% confidence intervals for the prediction of cognitive deficits by multiple combined digital biomarkers of the writing process, the MMSE scale, and the MoCA scale in the MCI and HC groups of the populations.

We assessed the presence of cognitive deficits in the MCI and HC groups through the participant’s work characteristic curve (ROC) combined with the above digital biomarkers of the writing process with intergroup variability, but considering the limited effective sample size of this study and the risk of model overfitting by including too many indicators, we used a stepwise regression method to downscale the digital biomarkers of the writing process, and after downscaling, average process pause time (*IPSDB*_8_), the number of pauses (*IPSDB*_10_), and variability in writing speed (*EFDB*_11_) were retained. We plotted the ROC curves for the discriminative ability of these three digital writing process biomarkers individually for HC and MCI groups, with an AUC of 0.783 (CI = 0.678–0.888) for the average process pause time (*IPSDB*_8_), an AUC of 0.675 (CI = 0.549–0.801) for the variability in writing speed (*EFDB*_11_), an AUC of 0.644 (CI = 0.516–0.773) for the number of pauses (*IPSDB*_10_). The joint AUC value of the above three writing process digital biomarkers was 0.918 (CI = 0.854–0.982), which was higher than the MMSE scale (AUC = 0.783, CI = 0.678–0.888) and MoCA scale (AUC = 0.859, CI = 0.772–0.947) are shown in Figures 7, 8.

## 4 Discussion

In this study, we propose a novel method for rapid intelligent screening of AD due to MCI based on the dynamic writing time series process. The method is based on a numerical writing assessment paradigm, which can characterize and quantify the cognitive deficits of MCI due to AD populations at a fine-grained level. We selected three digital biomarkers of the writing process, namely, average process pause time (*IPSDB*_8_), the number of pauses (*IPSDB*_10_), and variability in writing speed (*EFDB*_11_), whose combined warning efficacy was 0.918. This study provides a new and effective way to evaluate cognitive function of people with MCI due to AD based on human-computer interaction, and offers a new approach to rapid and intelligent screening of MCI due to AD. This method is not only time-consuming and low-cost, but also has a high early warning effect, which has an important practical application value.

Firstly, digital biomarkers based on the dynamic writing time series process can characterize executive function deficits in MCI populations at a fine-grained level. The results of the present study showed that stroke counts per minute (*EFDB*_3_) and writing efficiency (*EFDB*_4_) of the digital biomarkers of executive function in the MCI group were significantly smaller than those in the HC group, and the variability in writing speed (*EFDB*_11_) was greater than that in the HC group in both MCI groups, and these findings are basically in agreement with those of Yu and Chang (2019) and Li et al. (2023), which suggests the possibility of the existence of the populations in the MCI group with executive dysfunction. Previous studies have also shown that writing characteristics such as writing strokes and speed changes can be used as important indicators for assessing executive functioning in patients with MCI due to AD (An et al., 2023). Executive functioning is an umbrella term for a series of complex cognitive processes involved in the achievement of goal-directed behaviors by an individual, including a number of aspects such as planning, inhibitory control, and cognitive flexibility, which work synergistically with one another to ensure that an individual is able to plan effectively. These aspects work together to ensure that individuals can effectively plan, organize, monitor, and adjust their behaviors to achieve their goals (Diamond, 2013). Several studies have shown that executive dysfunction in people with MCI is primarily related to the frontal cortex, especially the dorsolateral prefrontal cortex (Liang et al., 2011; Wu et al., 2014). Numerous studies have shown that the dorsolateral prefrontal cortex plays a key role in a variety of executive functions, such as response inhibition, cognitive flexibility, working memory, planning, and abstract reasoning (Jung et al., 2022; Elliott, 2003; Hudziak et al., 2014). Functional magnetic resonance imaging (fMRI) studies showed that when MCI patients completed the Stroop range test and Wisconsin card classification tasks, the activation intensity and pattern of DLPFC were significantly abnormal, and their neural response time was significantly prolonged by Ko et al. (2009) and Miyake et al. (2000). Thus, MCI populations may suffer from executive dysfunction due to dysfunction in the dorsolateral prefrontal cortex, which ultimately manifests itself as abnormalities in numerical biomarkers of executive function during paradigm assessment.

Secondly, we found that digital biomarkers based on the dynamic writing time series can characterize the information processing speed of MCI populations at a fine-grained level. The results of this study showed that total task time (*IPSDB*_1_), task process time (*IPSDB*_2_), writing time (*IPSDB*_3_), total pause time (*IPSDB*_4_), initial pause time (*IPSDB*_5_), total process pause time (*IPSDB*_6_), maximum process pause time (*IPSDB*_7_), average process pause time (*IPSDB*_8_), variability in process pause time (*IPSDB*_9_), and the number of pauses (*IPSDB*_10_) were significantly higher in the HC group. These findings are in general agreement with those of Haworth et al. (2016) and Gonzales et al. (2017), and suggest a possible decline in information processing speed in the MCI populations. Previous studies have also shown that writing characteristics such as writing time and pause time can be important indicators for assessing information processing speed in MCI due to AD (Meulemans et al., 2022). Information processing speed is the speed at which an individual moves from receiving information to responding to or making decisions, and this metric plays an important role in cognitive functioning, learning ability, and performance in daily life. Studies have shown that slowing down the speed of information processing in patients with early AD may be related to a reduction in the volume of gray matter in the brain, especially atrophy of the hippocampus and entorhinal cortex (Toepper, 2017). Other studies have shown that the decrease in information processing speed in early AD patients is the result of coordinated degeneration of multiple brain regions, including cortical atrophy (frontal and parietal lobes), damage to the hippocampus and limbic system, default mode network dysfunction, and loss of white matter pathway integrity (Hassan et al., 2022; Van Dijk et al., 2012; Koch et al., 2015). Together, these changes lead to reduced neural signaling efficiency and abnormal allocation of cognitive resources. Therefore, the phenomenon of slowing down information processing speed in MCI populations during paradigm assessment ultimately manifests itself as an abnormality of numerical biomarkers of information processing speed during paradigm assessment process.

Then, previous studies have also used writing handwriting characteristics to screen for early warning MCI populations, but most of them are final outcome assessments without a fine-grained assessment of the dynamic process of writing time series, and they also suffer from poor age appropriateness and poor reliability with small sample sizes. The handwriting characteristics proposed by Jacek Kawa are good at distinguishing between patients with MCI, but they lack a detailed assessment of the writing process, and the task is relatively complex (Kawa et al., 2017). It requires a certain level of literacy and aids such as electronic pens, and is therefore somewhat unfavorable for large-scale screening. Nan-Ying Yu found that the handwriting accuracy of the MCI group differed significantly from that of the subjects in the normal group, but the sample size of the MCI and NC included was only 32, so the results needed to be further validated (Yu and Chang, 2019). The early warning performance of the new computational cognitive neural-based screening method for MCI due to AD in this study (AUC = 0.918) was improved compared to our previous fingertip interaction-based screening method (AUC = 0.830) (Li et al., 2023). In addition, the AUC of the MMSE and MoCA rating scales for early warning efficacy for MCI due to AD were 0.783 and 0.859, respectively, which still had shortcomings such as high subjectivity, long time-consumption, and low early warning efficacy compared with the new method of screening for MCI due to AD proposed in this study. Therefore, the digital written assessment paradigm in this study has the advantages of intelligence, convenience, and high early warning efficacy, which is worthy of further research and popularization. The results of the paradigm evaluation will be provided by professional physicians in combination with the patient’s clinical evaluation results and provide corresponding suggestions to reduce the potential impact of the paradigm evaluation results on the patient’s health. In addition, the digital writing evaluation paradigm designed in this study has been promoted and applied in many hospitals and communities in China. It is currently only used on desktop computers, and any laptops and desktop computers with touch functions can be evaluated for patients. Clinicians only need simple training to implement a digital patient assessment paradigm. In the future, we will expand this paradigm to platforms such as tablets and smartphones.

Finally, this study has some limitations. (1) Only 72 subjects were included in this study, with a small sample size. In the future, large-scale multi-center sample studies and cohort follow-up studies should be conducted, and multiple verification methods should be adopted, and machine learning methods should be considered to further improve the accuracy of the research results; (2) The dimensions of digital biomarkers extracted in this study were limited, and digital biomarkers in dimensions such as eye movement tracking, EEG, voice, and gait should be added to conduct a more comprehensive cognitive ability assessment; (3) The subjects of this study were limited to individuals who use the Chinese language, so the handwriting characteristics in this study may only be valid for Chinese speakers. However, this also limits universality across cultures and languages. In the future, we will discuss the adaptability of the paradigm to non-Chinese scripts, or how to replicate similar concepts in alphabetic language systems. (4) Although the early warning effect of digital biomarkers extracted in this study was high, the medical mechanism behind them was unknown. In the future, it is necessary to further verify their reliability and scientificity in combination with neuroimaging data such as fMRI and PET.

## 5 Conclusion

In summary, we propose the research hypothesis that digital biomarkers of the writing process are expected to portray cognitive deficits at a fine-grained level in MCI due to AD populations. Based on this hypothesis, we designed a writing digital assessment paradigm and extracted digital biomarkers that can characterize the writing process of the subjects to achieve fine-grained quantification of their cognitive behavioral processes. After clinical validation, the efficacy of digital biomarkers of writing process in distinguishing between AD-MCI and HC populations was up to 0.918, and this method provides ideas for early warning screening of AD-MCI and HC populations.

## Data Availability

The raw data supporting the conclusions of this article will be made available by the authors without undue reservation.
